# Mathematical Modeling of Heterogeneous Electrophysiological Responses in Human *β*-Cells

**DOI:** 10.1371/journal.pcbi.1003389

**Published:** 2014-01-02

**Authors:** Michela Riz, Matthias Braun, Morten Gram Pedersen

**Affiliations:** 1 Department of Information Engineering, University of Padua, Padua, Italy; 2 Alberta Diabetes Institute, Department of Pharmacology, University of Alberta, Edmonton, Alberta, Canada; University of Pittsburgh, United States of America

## Abstract

Electrical activity plays a pivotal role in glucose-stimulated insulin secretion from pancreatic 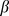-cells. Recent findings have shown that the electrophysiological characteristics of human 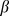-cells differ from their rodent counterparts. We show that the electrophysiological responses in human 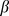-cells to a range of ion channels antagonists are heterogeneous. In some cells, inhibition of small-conductance potassium currents has no effect on action potential firing, while it increases the firing frequency dramatically in other cells. Sodium channel block can sometimes reduce action potential amplitude, sometimes abolish electrical activity, and in some cells even change spiking electrical activity to rapid bursting. We show that, in contrast to L-type 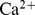 -channels, P/Q-type 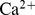 -currents are not necessary for action potential generation, and, surprisingly, a P/Q-type 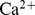 -channel antagonist even accelerates action potential firing. By including SK-channels and 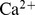 dynamics in a previous mathematical model of electrical activity in human 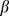-cells, we investigate the heterogeneous and nonintuitive electrophysiological responses to ion channel antagonists, and use our findings to obtain insight in previously published insulin secretion measurements. Using our model we also study paracrine signals, and simulate slow oscillations by adding a glycolytic oscillatory component to the electrophysiological model. The heterogenous electrophysiological responses in human 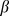-cells must be taken into account for a deeper understanding of the mechanisms underlying insulin secretion in health and disease, and as shown here, the interdisciplinary combination of experiments and modeling increases our understanding of human 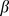-cell physiology.

## Author Summary

Insulin is a glucose-lowering hormone secreted from the pancreatic -cells in response to raised plasma glucose levels, and it is now well-established that defective insulin secretion plays a pivotal role in the development of diabetes. The -cells are electrically active, and use electrical activity to transduce an increase in glucose metabolism to calcium influx, which triggers insulin release. Experimental and theoretical studies on -cells from rodents have provided valuable insight in their electrophysiology. However, human -cells differ from their rodent counterparts in several aspects including their electrophysiological characteristics. We show that the electrophysiological responses in human -cells to a range of experimental manipulations are heterogeneous. We extend a previous mathematical model of electrical activity in human -cells to investigate such heterogeneous and nonintuitive electrophysiological responses, and use our findings to obtain insight in previously published insulin secretion measurements. By adding a glycolytic component to the electrophysiological model, we show that oscillations in glucose metabolism might underlie slow oscillations in electrical activity, calcium levels and insulin secretion observed experimentally. We conclude that the interdisciplinary combination of experiments and modeling increases our understanding of human -cell physiology and provides new insight in -cell heterogeneity.

## Introduction

Glucose-stimulated insulin secretion from human pancreatic 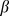-cells relies on the same major signaling cascade as their rodent counterparts, with electrical activity playing a pivotal role. Following metabolism of the sugar, ATP-sensitive potassium channels (K(ATP)-channels) close in response to the elevated ATP/ADP-ratio, which triggers action potential firing and 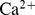 -influx through voltage-gated calcium channels. The resulting increase in intracellular calcium leads to insulin release by 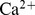 -dependent exocytosis [1]–[4]. However, the electrophysiological properties of human and rodent 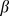-cells show important differences, e.g., with respect to their palette of expressed 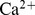 -channels and the role of 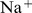 -channels, which contribute to electrical activity in human but not in rodent 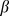-cells [1], [3].

Mathematical modeling has played important roles in studying the dynamics of electrical activity in rodent 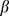-cells [5], [6], and could plausibly aid in understanding the electrophysiological responses in human 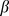-cells, and how they might differ from rodent cells. Recently, the first model of electrical activity in human 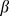-cells [7] was constructed from careful biophysical characterizations of ion channels in human 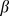-cells, mainly from Braun et al. [3]. The model [7] included 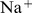 -channels, three types of 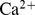 -channels, an unspecified leak-current, and several 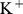 -channels: delayed rectifier (Kv) 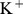 -channels, large-conductance (BK) 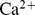 -sensitive 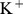 -channels, human ether-a-go-go (HERG) 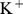 -channels as well as K(ATP)-channels. Recently evidence for small conductance (SK) 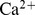 -sensitive 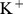 -channels in human 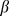-cells was published [4], [8], a current not included in the mathematical model [7].

The model [7] was shown to reproduce, depending on parameter values, spiking or rapid bursting electrical activity, which could be modified in accordance with a series of experiments by simulating pharmacological interventions such as ion channel blocking. These experiments were in general straightforward to interpret, also without a model. For example, the facts that blocking depolarizing 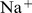 - or 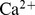 -currents slowed or abolished electrical activity [3] are as one would expect.

Here, we extend the previous model for human 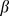-cells [7] by including SK-channels and 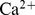 dynamics, and show that the model now has reached a level of maturity that allows us to get insight in less intuitive experimental findings. We find experimentally that SK-channels in some cells play an important role in controlling electrical activity, while they have virtually no effect in other cells. Using the extended version of the model, we show that this difference can be explained by differences in the excitability of the cells. Moreover we find that SK-channels can substitute for HERG-channels in controlling rapid bursting. We also show that blocking 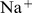 -channels in some cells can transform spiking behavior into rapid bursting, in contrast to the usual effect of 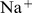 -channel blockers, which in general reduce or abolish spiking behavior [3], [9], [10]. Using our model we suggest that this happens in cells with a large 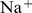 -current and that BK-channels play a prominent role. In addition, we suggest that SK-channels might underlie the surprising result that blocking depolarizing P/Q-type 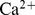 -channels *enhances* electrical activity, in contrast to the effect of L- or T-type 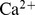 -channel antagonists, which reduce excitability and electrical activity [3]. Our model is then used to investigate paracrine effects of 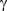-aminobutyric acid (GABA) and muscarinic signaling on electrical activity. Finally, we show experimentally slow oscillations in electrical activity that might underlie pulsatile insulin secretion from human pancreatic islets, and by adding an oscillatory glycolytic component [11] to the electrophysiological model, we simulate such slow bursting patterns.

## Results

To investigate a series of experimental observations, we have extended our previous model of electric activity in human 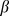-cells [7] by including several additional components of human 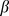-cell physiology, as described in the following, and in greater details in the Methods section. The mathematical modeling was carefully based on experimental data, as was the development of the core electrophysiological part modeled previously [7]. The extended model includes small conductance 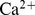 -activated 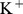 -channels (SK-channels), which are expressed in human 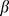-cells [4], [8]. The size of the SK-current was estimated from experimental measures [8]. We made a special effort to carefully model the submembrane dynamics of 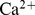, since SK-channels are controlled by the submembrane 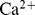 concentration (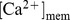), which reacts rapidly to each action potential so that activation of SK-channels might influence the generation and shape of action potentials during spiking electrical activity. In order to study paracrine signalling, our extended model also includes currents due to 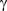-aminobutyric acid (GABA) activation of GABA*_A_* receptors, which are ligand-gated 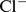 channels operating in human 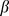-cells [12]. Finally, a glycolytic oscillator [11] has been added to the model to account for slow oscillations in ATP levels in human 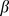-cells [13], [14], which have been suggested to underlie slow patterns of electrical activity, 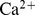 oscillations, and pulsatile insulin release.

Summarizing, the new version of the model now includes components from glucose metabolism, additional electrophysiological components (SK-channels and GABA*_A_* receptors), and 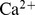 dynamics, leading to a global model of human 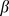-cell physiology, which, importantly, is based as far as possible on published data from human 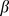-cells.

### SK channels

**Figure 1:**
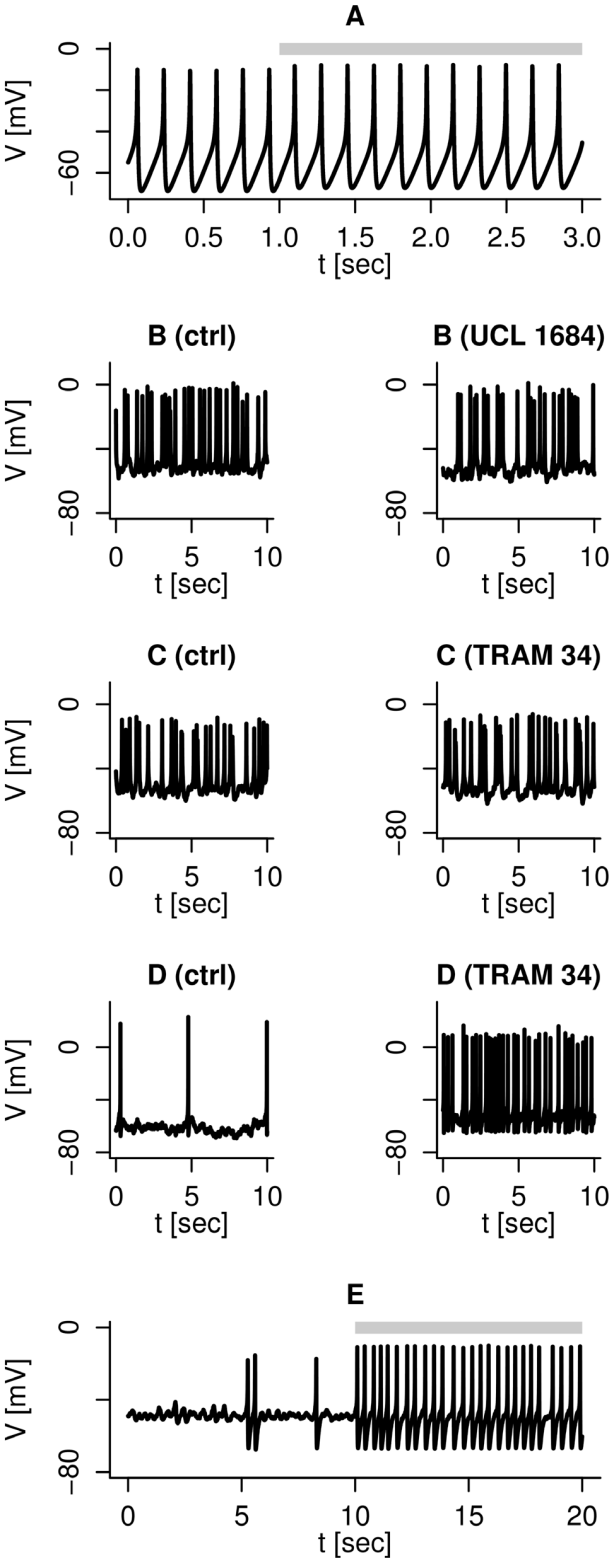
Heterogenous responses to SK-channel block. Note the differences in time-scales. A: Simulation, with default parameters, showing no effect of SK-channels block (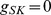 nS/pF during the period indicated by the gray bar). B: Experimental recording of spiking electrical activity in the same human 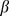-cell before (left) and during (right) application of the SK1-3 channel antagonist UCL-1684 (0.2 µM). C: Experimental recording of spiking electrical activity in the same human 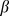-cell before (left) and during (right) application of the SK4 channel antagonist TRAM-34 (1 µM), which had little effect on the action potential frequency in this cell. D: Experimental recording of spiking electrical activity in the same human 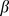-cell before (left) and during (right) application of the SK4 channel antagonist TRAM-34 (1 µM), which accelerated the action potential frequency in this cell. E: Stochastic simulation reproducing the dramatic effect of SK-channels block (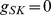 nS/pF during the period indicated by the gray bar). Other parameters took default values, except 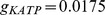 nS/pF.

When stimulated by glucose, human 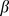-cells show electrical activity [1], [3]. Human 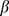-cells express SK-channels [4], [8], which might participate in controlling electrical activity. To study the role of SK-channels in human 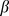-cells, we included SK-channels and 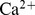 dynamics in our previous model [7]. The new model with standard parameters produces spiking electrical activity (Fig. 1A), which is virtually unaffected by setting the SK-conductance 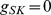 nS/pF simulating SK-channels block. This model prediction was confirmed by our experimental data, and was also observed in at least one cell by Jacobson et al. [8]. Fig. 1B shows an example of spiking electrical activity in a human 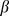-cell stimulated by 6 mM glucose, where addition of the SK1-3 channel blocker UCL 1684 (0.2 µM) did not affect the spiking pattern. Unchanged or marginal effects on electrical activity were also seen with a specific SK4 channel antagonist, TRAM-34 (1 µM, Fig. 1C). However, in some cells TRAM-34 application increased the action potential dramatically (Fig. 1D) in agreement with observations with the SK-channel antagonist apamin [8]. Note that before SK-channel block, the cell in Fig. 1D was almost quiescent, and fired action potentials very infrequently and randomly. This increase in spike frequency can be simulated by a stochastic version of the model. By including noise in the K(ATP) current, an otherwise silent cell produces infrequent action potentials evoked by random perturbations (Fig. 1E). When the SK-conductance is set to 0 nS/pF, the cell starts rapid action potential firing driven by the underlying deterministic dynamics. The model analysis indicates that this mechanism only works if the cell is very near the threshold for electrical activity in the absence of the SK-channel antagonist. Düfer et al. [15] suggested a similar, important role for SK4 channels in promoting electrical activity in murine 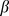-cells at subthreshold glucose concentrations. Summarizing, cell-to-cell heterogeneity can explain the differences seen in the electrophysiological responses to SK-channel antagonists.

**Figure 2:**
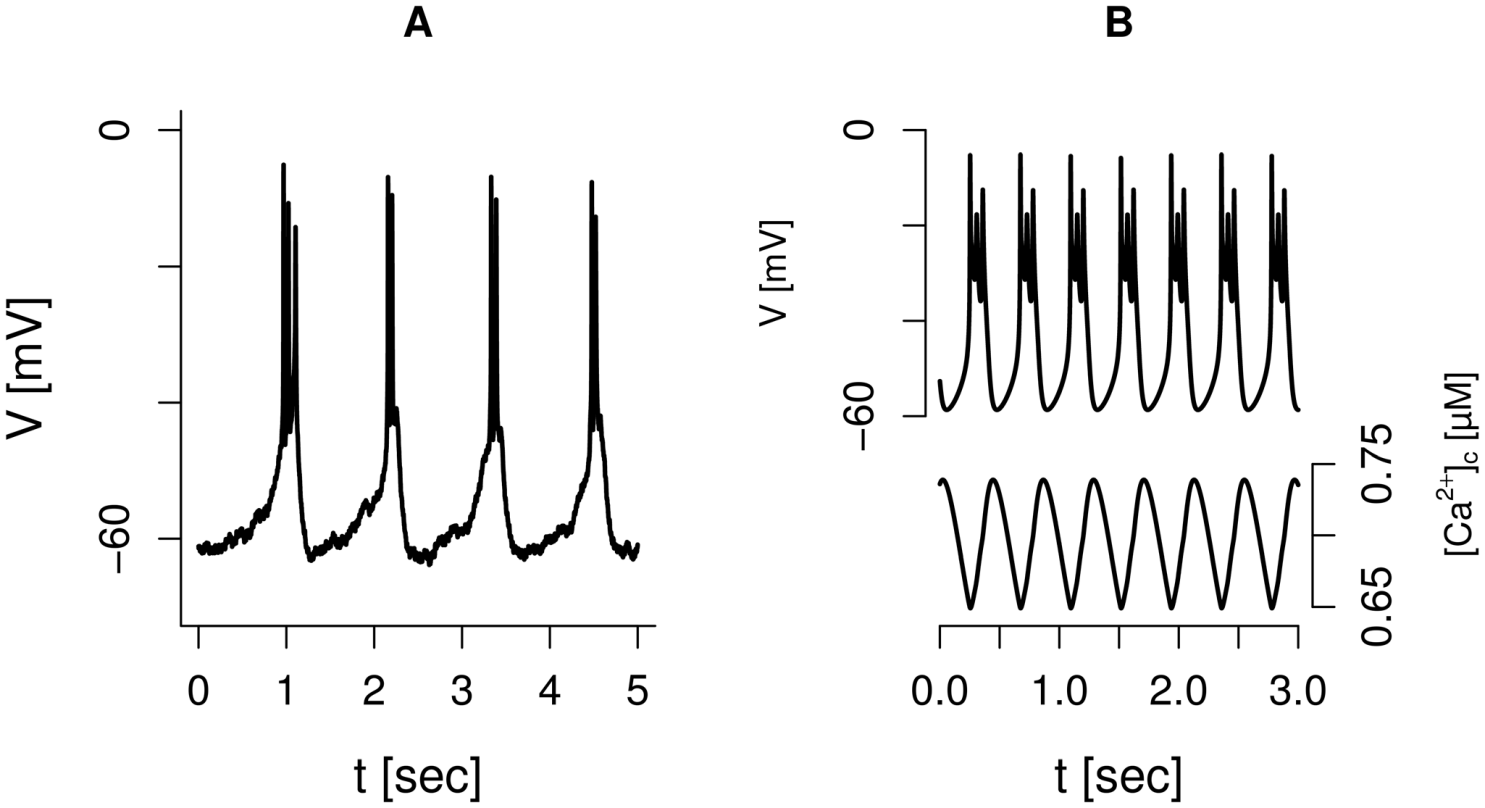
Bursting in human 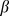-cell. A: Experimental recording of rapid bursting in a human 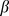-cell. B: Simulation of bursting driven by 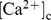 via SK-channels. Default parameters except 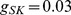 nS/pF, 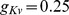 nS/pF, 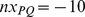 mV.

In addition to spiking electrical activity, human 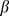-cells often show rapid bursting, where clusters of a few action potentials (active phases) are separated by hyperpolarized silent phases [1], [4], [9], [10], [16] (Fig. 2A). The extended model presented here can also reproduce this behavior (Fig. 2B) as could the previous version of the model [7], where the alternations between silent and active phases were controlled by HERG-channels. In contrast, in the present version of the model the rapid burst pattern (Fig. 2B, upper trace) can be controlled by SK-channels, which in turn are regulated by 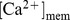 and ultimately by bulk cytosolic 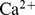 levels (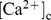). The simulated cytosolic 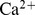 concentration shows the characteristic sawtooth pattern (Fig. 2B, lower trace) of a slow variable underlying bursting [17], [18]. Thus, as in the pioneering model by Chay and Keizer [19], 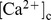 increases during the active phase and activates SK-channels, which eventually repolarize the cell. During the silent phase 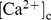 decreases and SK-channels close, allowing another cycle to occur.

### 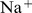 channels

**Figure 3:**
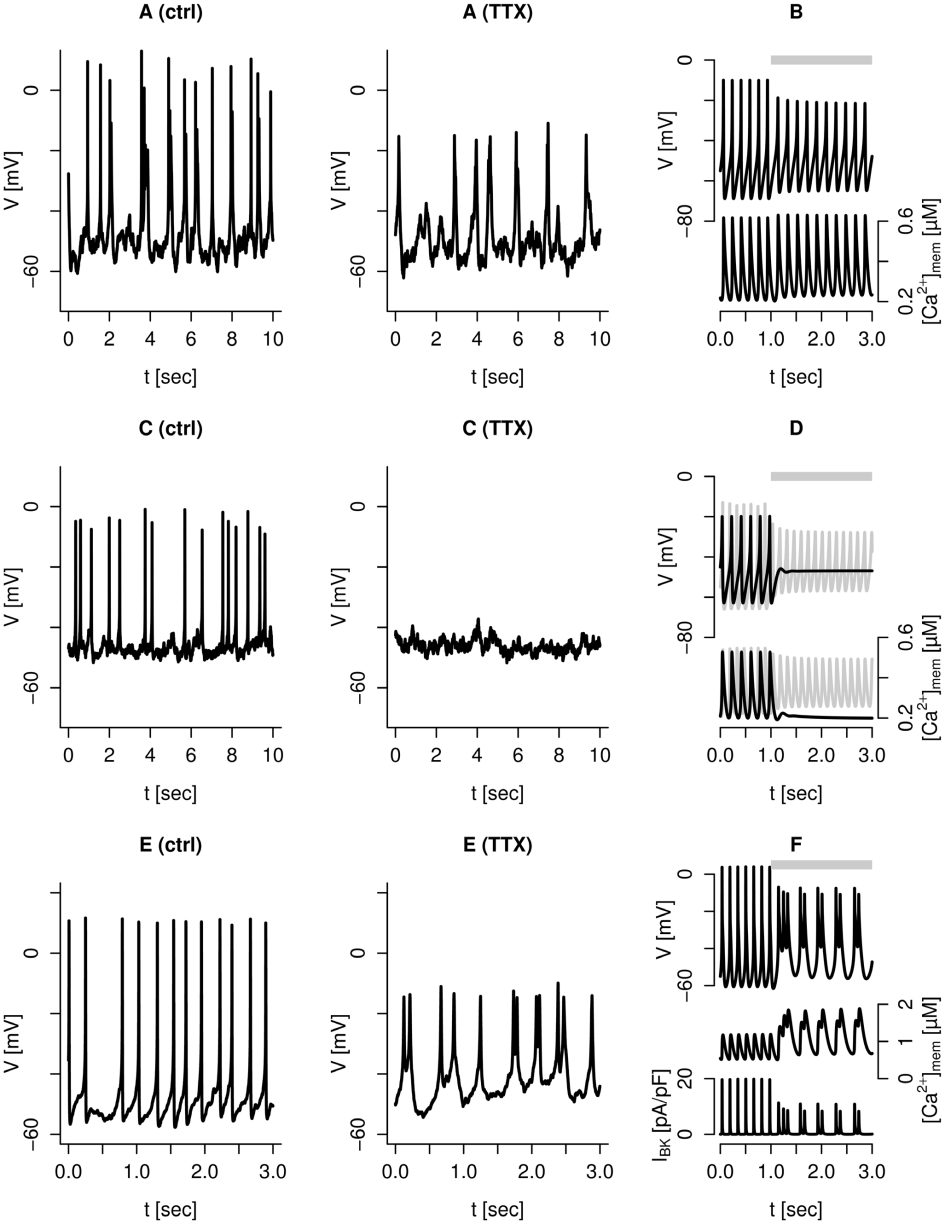
Tetrodotoxin (TTX, 0.1 µg/ml) has different effects on electrical activity in human 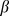-cells. A: TTX caused a reduction in action potential amplitude in this human 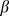-cell. B: Simulation with default parameters showing V (upper trace, left axis) and 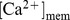 (lower trace, right axis), reproducing the data in panel A. C: TTX abolished action potential firing in this human 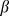-cell. D: Simulations of V and 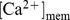 with default parameters except 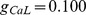 nS/pF. With default K(ATP)-channel conductance 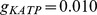 nS/pF, the simulation reproduces the data in panel C (black traces). When 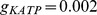 nS/pF, the model shows continued firing with 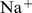 -channel block (gray traces). E: TTX changed spiking into rapid bursting electrical activity in this human 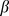-cell. F: Simulation showing V (upper), 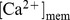 (middle), and 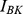 (lower), reproducing the data in panel E. Parameters took default values, except 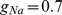 nS/pF, 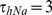 ms, 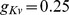 nS/pF, 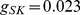 nS/pF, 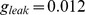 nS/pF, and 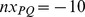 mV. The extracellular glucose concentration was 6 mM in all experiments. Each couple of experimental traces (panels A, C and E) is from the same human 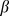-cell before (left) and during (right) application of TTX. In the simulations, the 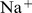 -channel conductance 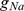 was set to 0 nS/pF during the period indicated by the gray bars.

Blocking voltage-dependent 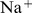 -channels in human 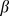-cells showing spiking electrical activity with tetrodotoxin (TTX) typically reduces the action potential amplitude by ∼10 mV, and broadens its duration [3], [9], [10] (Fig. 3A). The previous version of the model [7] could reproduce these results, though the reduction in peak voltage was slightly less than observed experimentally. The inclusion of SK-channels in the model leads to a greater reduction in the spike amplitude (Fig. 3B, upper trace) when 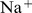 -channels are blocked. This improvement is because of a mechanism where the slower upstroke in the presence of 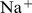 -channel blockers allows submembrane 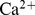 to build up earlier and to higher concentrations (Fig. 3B, lower trace), and consequently to activate more SK-channels, which in turn leads to an earlier repolarization reducing the action potential amplitude. In other experiments (Fig. 3C) [16], TTX application suppresses action potential firing. In agreement, simulated spiking electrical activity can be suppressed by TTX application if the cell is less excitable because of, for example, smaller 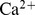 -currents (Fig. 3D, upper, black trace). Before TTX application, the simulated cell had less hyperpolarized inter-spike membrane potential (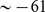 mV; Fig. 3D) compared to the simulation with default parameters (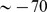 mV, Fig. 3B). This finding is in accordance with experimental recordings (compare Fig. 3A and 3C). The cessation of action potential firing leads to a reduction in simulated 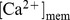 (Fig. 3D, lower, black trace). The model predicts that spiking, electrical activity can continue in presence of TTX even in less excitable cells, e.g., with lower depolarizing 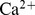 -currents, if the hyperpolarizing K(ATP)-current is sufficiently small (Fig. 3D, upper, gray trace). In this case, 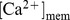 is nearly unchanged (Fig. 3D, lower, gray trace). Hence, it is the relative sizes of the depolarizing and hyperpolarizing currents that determine whether TTX application silences the cell or allows the cell to remain in a region where action potential firing continues. The model thus predicts that in some cells, which stop firing action potentials in the presence of TTX, increased glucose concentrations or sulfonylureas (K(ATP)-channel antagonists) could reintroduce spiking electrical activity.

More surprisingly, TTX application can change spiking electrical activity to rapid bursting in some cells (Fig. 3E). This behavior can also be captured by the model (Fig. 3F). To simulate this behavior it was necessary to increase the size of the 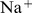 -current. Without TTX, the big 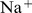 -current leads to large action potentials, which activate sufficient BK-current to send the membrane potential back to the hyperpolarized state, allowing a new action potential to form. With 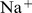 -channels blocked, there is insufficient depolarizing current to allow full action potentials to develop. In consequence, less BK-current is activated (Fig. 3F, lower trace), and the membrane potential enters a regime with more complex dynamics where smaller spikes appear in clusters from a plateau of ∼−40 mV. The change to bursting activity leads to a notable increase in simulated 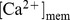 (Fig. 3F, middle trace).

### 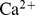 channels

**Figure 4:**
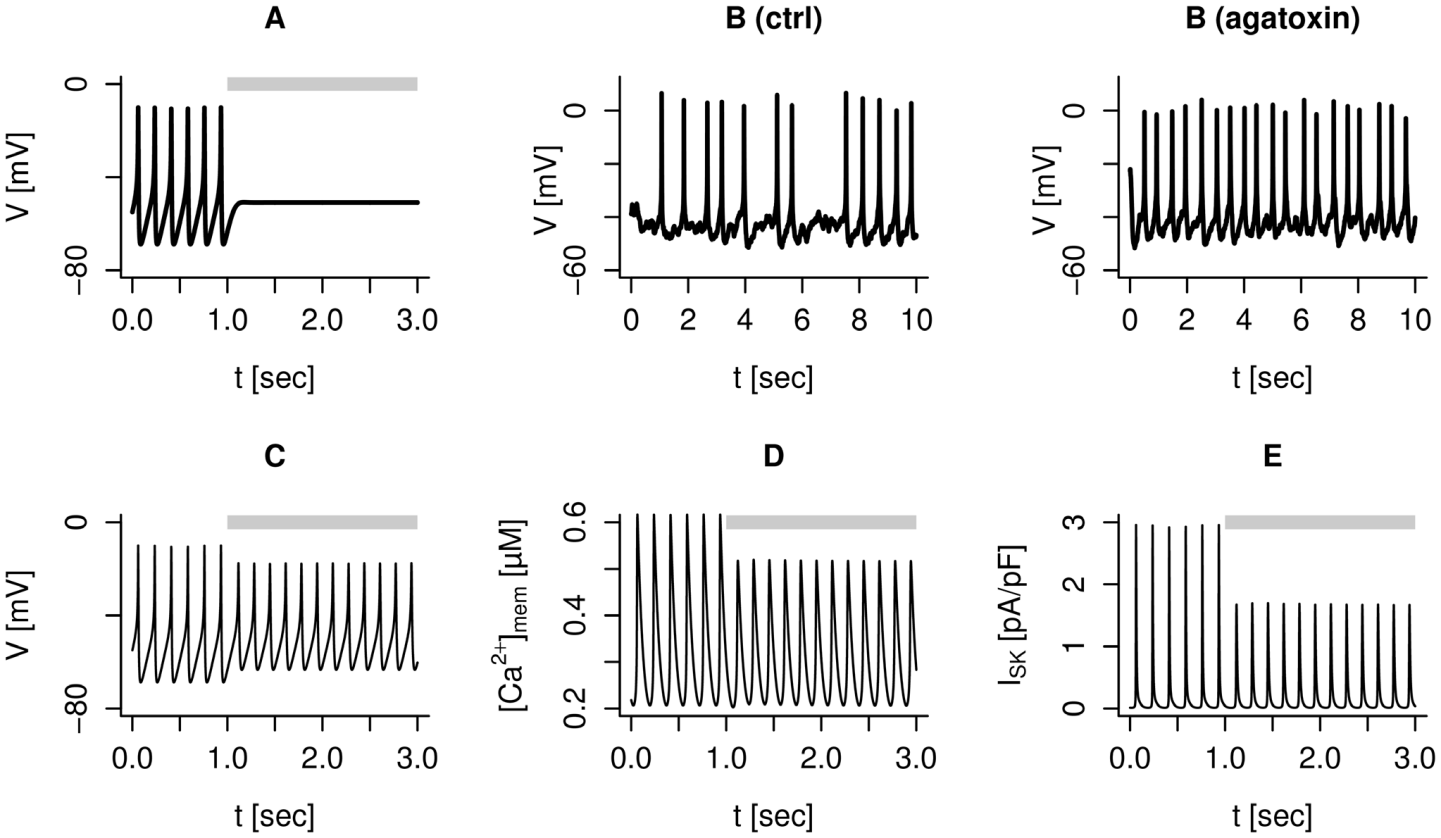
Block of L- and P/Q-type 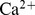 -channels affects electrical activity differently. A: Spiking electrical activity is suppressed by L-type 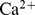 -channel block in the model with default parameters, and 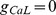 nS/pF during the period indicated by the gray bar. B: Spiking electrical activity is accelerated by the application of 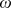-agatoxin IVA in human 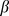-cells. Recordings from the same human 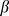-cell in 6 mM extracellular glucose before (left) and during (right) application of 200 nM 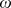-agatoxin IVA. C: Model simulation with default parameters of the membrane potential during spiking electrical activity under control conditions and after blockage of P/Q-type 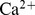 -channels (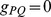 nS/pF during the period indicated by the gray bar). D: In the model, the peak submembrane 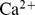 -concentration 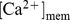 is lower when P/Q-type channels are blocked. E: The reduced 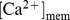 activate less SK-current when P/Q-type channels are blocked.

High-voltage activated L- and P/Q-type 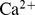 -currents are believed to be directly involved in exocytosis of secretory granules in human 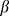-cells [1], [3], [4], [20], [21]. Blocking L-type 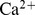 -channels suppresses electrical activity [3], which is reproduced by the model (Fig. 4A) [7], and the lack of electrical activity is likely the main reason for the complete absence of glucose stimulated insulin secretion in the presence of L-type 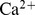 -channel blockers [3]. Thus, L-type 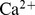 -channels participate in the upstroke of action potentials and increases excitability of human 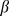-cells.

In contrast, and surprisingly, application of the P/Q-type 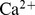 -channel antagonist 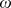-agatoxin IVA does not block or slow down electrical activity, but leads to an *increased* spike frequency (Fig. 4B). Electrical activity continues also in our model simulations of P/Q-type channel block with slightly increased spike frequency (Fig. 4C). Reduced 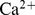 entry leads to lower peak 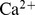 concentrations in the submembrane space (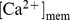; Fig. 4D). As a consequence, less hyperpolarizing SK-current is activated (Fig. 4E), which leads to an increase in spike frequency (Fig. 4C). Hence, the reduction in excitability caused by blockage of the P/Q-type 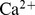 -current can be overruled by the competing increase in excitability due to the smaller SK-current. Experimentally, 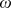-agatoxin IVA application reduced the action potential amplitude slightly in 3 of 4 cells (by 2.0–4.3 mV), a finding that was quantitatively reproduced by the model, although the reduction was larger (∼7.5 mV in Fig. 4C). A direct conclusion from Fig. 4B is that the P/Q-type 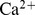 -current is not needed for the action potential upstroke, unlike the L-type current, probably because of the fact that P/Q-type channels activate at higher membrane potentials than L-type channels. The fact that electrical activity persists with P/Q-type 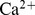 -channels blocked, albeit with lower peak 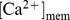, could underlie the finding that 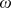-agatoxin IVA only partly inhibits insulin secretion [3].

### Paracrine effects on electrical activity

**Figure 5:**
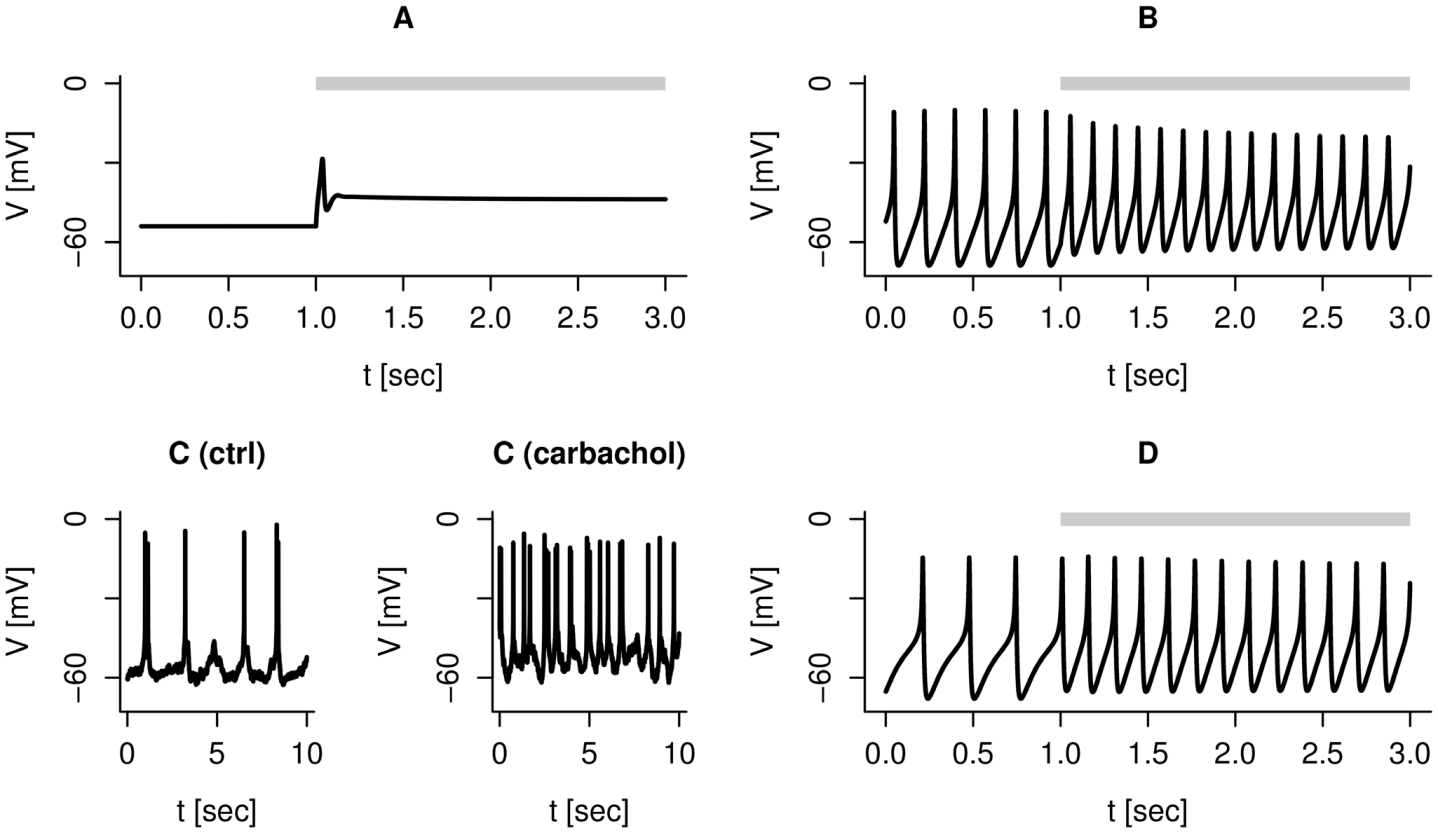
Paracrine effects on electrical activity. A: Simulation of application of 100 µM GABA to a silent cell (reproducing Fig. 7A in [12]). Default parameters except 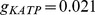 nS/pF. GABA application was simulated by setting 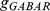 to 0.1 nS/pF during the period indicated by the gray bar. B: Simulation of application of 10 µM GABA to an active cell (reproducing Fig. 7B in [12]). GABA application was simulated by setting 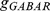 to 0.020 nS/pF during the period indicated by the gray bar. Other parameters took default values. C: Experimental recording of spiking electrical activity in the same human 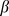-cell before (left) and during (right) application of carbachol (20 µM). D: Simulation of accelerated action potential firing due to carbachol application. Default parameters except 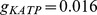 nS/pF. Carbachol application was simulated by increasing 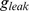 to 0.030 nS/pF during the period indicated by the gray bar.

The neurotransmitter 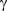-aminobutyric acid (GABA) is secreted from pancreatic 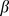-cells, and has been shown to stimulate electrical activity in human 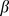-cells [12]. In human 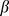-cells, GABA activates GABA*_A_* receptors, which are ligand-gated 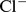 channels, thus creating an additional current. Notably, the 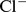 reversal potential in human 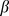-cells is less negative than in many neurons, and positive compared to the 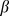-cell resting potential, which means that 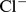 currents, such as the GABA*_A_* receptor current, stimulate action potential firing in 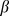-cells. Hence, GABA is a excitatory transmitter in 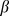-cells, in contrast to its usual inhibitory role in neurons. We simulate the addition of GABA by raising the GABA*_A_* receptor conductance. In a silent model cell with a rather large K(ATP)-conductance, simulated GABA application leads to a single action potential whereafter the membrane potential settles at ∼−45 mV (Fig. 5A), in close correspondence with the experimental results [12]. In an active cell, the simulation of activation of GABA*_A_* receptors leads to a minor depolarization and increased action potential firing (Fig. 5B), as found experimentally [12].

Another neurotransmitter, acetylcholine, might also play a paracrine role in human pancreatic islets, where it is released from 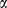-cells, and activates muscarinic receptors in 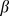-cells [22]. Muscarinic receptor activation by acetylcholine triggers a voltage-insensitive 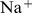 -current in mouse pancreatic beta-cells [23], and similarly, the muscarinic agonist carbachol activates nonselective 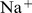 leak channels (NALCN) in the MIN6 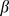-cell line [24]. Based on these findings, it was speculated that muscarinic activation of NACLN currents in human 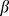-cells might participate in the positive effect of acetylcholine and carbachol on insulin secretion [4]. Experimentally, we found that carbachol (20 µM) accelerates action potential firing (Fig. 5C). We tested the hypothesis of a central role of leak current activation by increasing the leak conductance in the model to simulate carbachol application, which caused accelerated action potential firing. The simulation thus reproduced the experimental data, and lends support to the hypothesis that carbachol and acetylcholine can accelerate action potential firing via muscarinic receptor-dependent stimulation of NALCN currents [4].

### Slow oscillations

**Figure 6:**
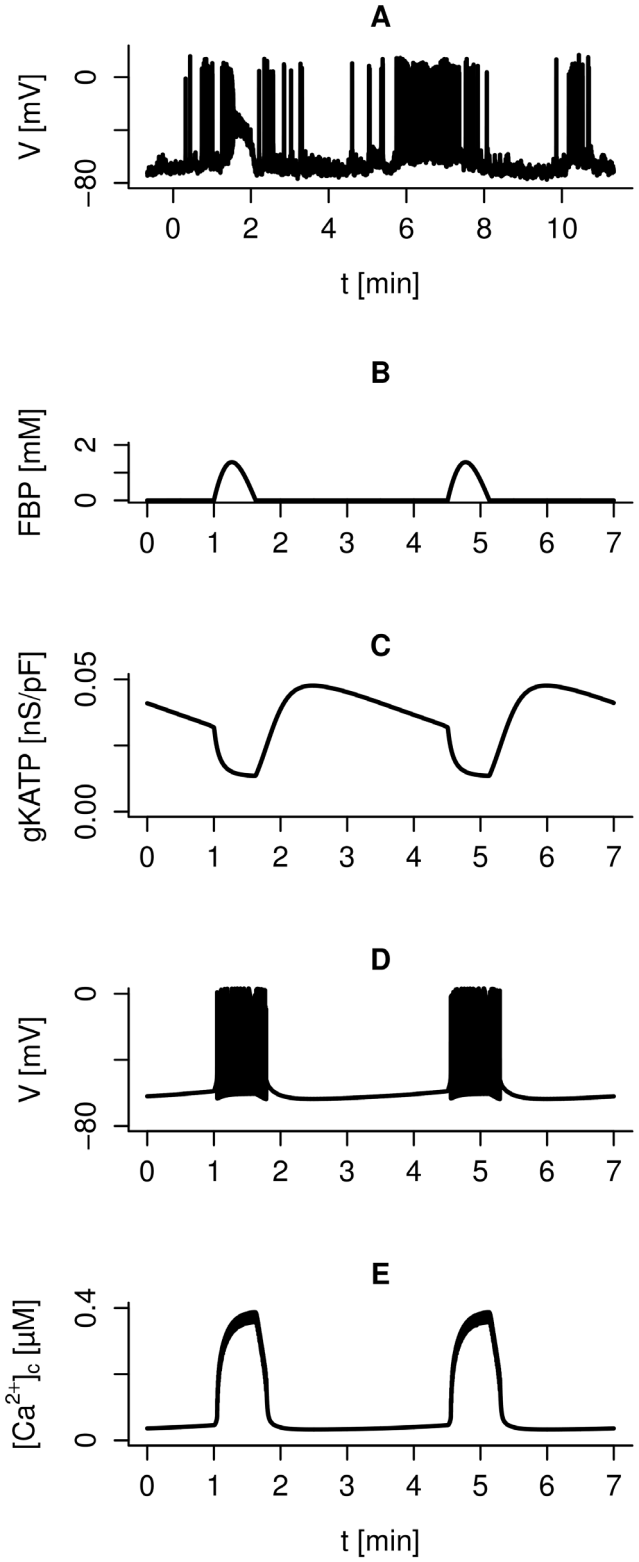
Metabolically driven slow waves of electrical activity and 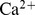 oscillations. A: Experimental recording of slow oscillations in action potential firing in a human 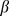-cell exposed to 10 mM glucose. B–D: Simulation of slow bursting driven by glycolytic oscillations with glucose concentration 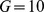 mM and default parameters, except 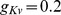 nS/pF, 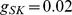 nS/pF, 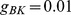 nS/pF. Oscillations in glycolysis create pulses of FBP (B), which via ATP production modulates K(ATP) channels in a periodic fashion (C). The rhythmic changes in K(ATP) conductance drives slow patterns of electrical activity (D), which causes oscillations in the intracellular 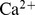 concentration (E).

We finally use our model to address the origin of slow rhythmic patterns of electrical activity in human 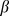-cells (Fig. 6A) [4], [25], which likely underlie slow oscillations in intracellular 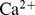 [26], [27] and pulsatile insulin release [28], [29]. Based on accumulating evidence obtained in rodent islets [5], [30], we have previously speculated that oscillations in metabolism could drive these patterns [7]. In support of this hypothesis, oscillations in ATP levels with a period of 3–5 minutes have been observed in human 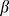-cells [13], [14]. By adding a glycolytic component [11], which can oscillate due to positive feedback on the central enzyme phosphofructokinase (PFK), our model can indeed simulate such periodic modulation of the electrical pattern, where action potential firing is interrupted by long silent, hyperpolarized periods, which drives slow 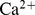 oscillations (Fig. 6).

## Discussion

Human 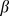-cells show complex and heterogeneous electrophysiological responses to ion channel antagonists. It can therefore sometimes be difficult to reach clear conclusions regarding the participation of certain ion channels in the various phases of electrical activity, in particular since some of the electrophysiological responses are nonintuitive as shown here. A deeper understanding of the role of ion channels in electrical activity and insulin secretion could have important clinical benefits, since it might help in the development of new anti-diabetic drugs.

We have here shown how mathematical modeling can help in interpreting various electrophysiological responses, and in particular, to study the effect of competing effects and cell heterogeneity. The role of SK-channels in human 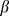-cells is still not clear. We (Fig. 1) and others [8] have found heterogeneous electrophysiological responses to SK-channel antagonists. Our model suggests that these differences can be caused by underlying variations in cell excitability: Less excitable 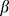-cells that produce action potentials evoked mostly by stochastic channel dynamics show a clear increase in action potential frequency when SK-channels are blocked (Fig. 1DE). In contrast, spiking electrical activity in very active cells is driven by the deterministic dynamics caused by ion channel interactions, and is nearly unchanged by SK-channel blockers (Fig. 1A–C). We showed also that rapid bursting activity can be driven by 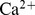 and SK-channels (Fig. 2), which could add a complementary mechanism to HERG-channel dynamics [7] for the control of rapid bursting.

The wide range of responses to TTX could be accounted for by a single model but with different parameters, i.e., differences in the relative size of the various currents. A peculiar finding is the qualitative change from spiking to rapid bursting seen in some cells (Fig. 3E). We suggest that this happens in human 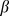-cells with large 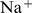 -currents. The blockage of this depolarizing current reduces the amplitude of the action potentials, and as a consequence, the size of the hyperpolarizing BK-current. Under the right conditions, the combination of these competing events allows the membrane potential to enter a bursting regime controlled by SK- and/or HERG-channels (Fig. 3F). Interestingly, it has been found that TTX reduces insulin secretion evoked by 6 mM glucose greatly, but at glucose levels of 10–20 mM, the effect of TTX on secretion is smaller [3], [9], [10]. Based on our simulations showing that less excitable cells cease to fire in the presence of TTX (Fig. 3D, black traces), but that lower 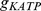 can reintroduce spiking activity (Fig. 3D, gray traces), we suggest that at low, near-threshold glucose levels TTX abolishes electrical activity in many cells, which reduces the 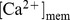 and consequently insulin secretion greatly (Fig. 3D, black traces). At higher glucose concentrations, 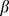-cells have lower K(ATP)-conductance and in some of the cells that stop firing in low glucose concentration the effect of TTX on electrical activity and 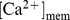 is smaller (Fig. 3D, gray traces). Hence, more 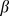-cells remain active in the presence of TTX at high than at low glucose levels. Consequently, insulin secretion is more robust to TTX at higher glucose concentrations.

Similarly, insulin release is more affected by the P/Q-type 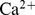 -channel blocker 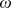-agatoxin IVA at 6 mM (−71%) than at 20 mM (−31%) glucose [3]. This is in contrast to L-type 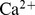 -channel antagonists, which abolish insulin secretion at both high (15–20 mM) and low (6 mM) glucose concentrations [1], [3], [10]. These results concerning L-type 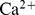 -channel block are easily explained by the fact that L-type channel activity is necessary for action potential generation [3] (Fig. 4A). In contrast, we showed that electrical activity in human 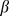-cells not only persists, but is accelerated by 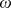-agatoxin IVA (Fig. 4B). The counter-intuitive finding of increased excitability and electrical activity when the depolarizing P/Q-type 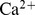 -current is blocked by 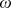-agatoxin IVA can be accounted for by an even greater reduction in the hyperpolarizing SK-current due to reduced 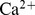 -influx and consequently lower 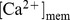.

Our mathematical modeling confirmed that GABA released from human 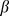-cells can have a role as a positive feedback messenger. GABA application has been shown to depolarize both silent and active human 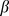-cells [12], which was reproduced here. A detailed characterization of GABA*_A_* receptor currents would refine the analysis presented here.

Data from mouse 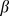-cells [23] and the MIN-6 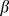-cell line [24] suggest that muscaric agonists such as carbachol and acetylcholine stimulate insulin secretion partly by activating NACLN currents. Using our model we could translate this finding to the human scenario, thus testing the hypothesis that this mechanism is also operating in human 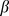-cells [4]. Our simulations confirmed that increased leak currents can underlie the change in electrical activity found experimentally (Fig. 5C). The incretin hormone glucagon-like peptide 1 (GLP-1) has also been shown to act partly via activation of leak channels [31], a mechanism which might be involved in activating otherwise silent 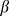-cells [7], [32], [33]. These results suggest that leak currents could play important roles in controlling electrical activity in 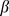-cells, and potentially be pharmacological targets. Further studies are clearly needed to investigate these questions.

We were also able to simulate slow rhythmic electrical activity patterns by adding an oscillatory glycolytic component to the model. To date, there is to our knowledge no evidence of oscillations in glycolytic variables in human (or rodent) 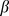-cells or islets, but ATP levels have been found to fluctuate rhythmically also in human 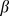-cells [13], [14], supporting the idea of metabolism having a pacemaker role. In agreement, data from rodent 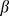-cells show accumulating evidence for oscillations in metabolism playing an important role in controlling pulsatile insulin secretion [5], [30]. It will be interesting to see if these findings in rodents are applicable to human 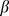-cells.

Regarding the model development, the inclusion of SK-channels in the model provided insight that was not within reach with the previous version of the model [7]. Besides the direct investigation of the role of SK-channels, the acceleration in action potential firing seen with P/Q-type 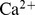 channel blockers (Fig. 4) can not be reproduced by the older version of the model without SK-channels [7]. Moreover, considering the effect of TTX on spike amplitude, a better correspondence between experiments and simulations was found with SK-channels included in the model. To model SK-channel activation accurately, we made a special effort to describe 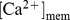 carefully. Submembrane 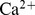 responds rapidly to an action potential, while 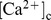 integrates many action potentials. The rapid submembrane dynamics has important consequences for the study of the role of SK-channels in spiking electrical activity, e.g., it was crucial for explaining the larger effect of TTX on spike amplitude in this version of the model. Most models of electrical activity in rodent 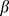-cells do not include a submembrane 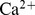 compartment, but these models were typically built to explain the slow bursting patterns seen in rodent islets with a period of tens of seconds. For these long time scales, the rapid dynamics in the submembrane compartment is not important. In contrast, the situation is different in human 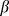-cells with their faster dynamics.

## Methods

### Modeling

We build on the previously published Hodgkin-Huxley type model for human 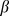-cells [7], which was mainly based on the results of Braun et al. [3], who carefully assured that investigated human islet cells were 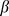-cells. We include SK-channels in the model. Since these channels are 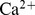 -sensitive and located at some distance from 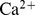 -channels [34] we also model 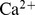 -dynamics in a submembrane layer controlling SK-channel activity.

The membrane potential 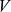 (measured in mV) develops in time (measured in ms) according to
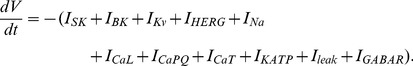
(1)All currents (measured in pA/pF), except the SK-current 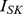 and the GABA*_A_* receptor mediated current 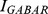, are modeled as in [7]. Expressions and parameters are given below. For the stochastic simulation in Fig. 1E, we included “conductance noise” [35] in the K(ATP) current by multiplying 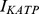 by a stochastic factor 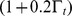, where 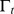 is a standard Gaussian white-noise process with zero mean and mean square 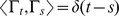, see also [36]–[38].

SK-channels are assumed to activate instantaneously in response to 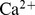 elevations at the plasma membrane but away from 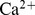 channels [34], and are modeled as [39]
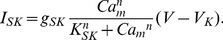
(2)In human 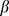-cells, flash-released 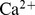 triggered a ∼10 pA current at a holding current of 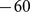 mV, presumably through SK-channels [8]. Assuming that SK-channels were nearly saturated by 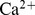, the maximal SK-conductance is estimated to be 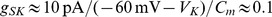 nS/pF. Here, 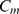 = 10 pF is the capacitance of the plasma membrane [3].

In Eq. 2, 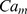 is the submembrane 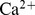 concentration (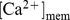; measured in µM), which is described by a single compartment model [21]
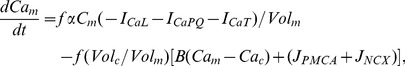
(3)where 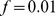 is the ratio of free-to-total 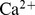, 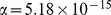 µmol/pA/ms changes current to flux, and 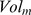 and 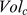 are the volumes of the submembrane compartment and the bulk cytosol, respectively. 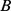 describes the flux of 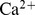 from the submembrane compartment to the bulk cytosol, 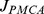 is the flux through plasma membrane 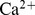 -ATPases, and 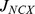 represents 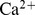 flux through the 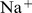 - 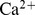 exchanger. Cytosolic 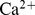 (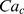; measured in µM) follows
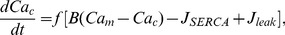
(4)where 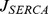 describes SERCA pump-dependent sequestration of 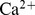 into the endoplasmic reticulum (ER), and 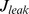 is a leak flux from the ER to the cytosol. Expressions and parameters for the 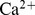 fluxes are taken from [40].

The submembrane compartment volume is estimated based on the considerations of Klingauf and Neher [41], who found that a shell model (in contrast to a domain model) describes submembrane 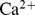 satisfactorily when the shell-depth is chosen correctly. The 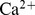 dynamics between channels can be estimated from a shell model at a depth of ∼23% of the distance to a 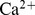 -channel. In mouse 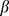-cells the interchannel distance has been estimated to be 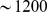 nm [42]. Moreover, SK-channels are located 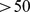 nm from 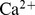 channels [34].

Based on these considerations, we modeled the submembrane space controlling SK-channels as a shell of depth 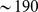 nm. The radius of a human 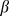-cell is 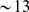 µm, which gives cell volume (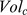), shell volume (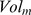) and internal surface area (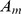) of the shell, of
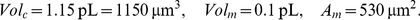
(5)The flux-constant 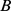 can then be calculated as [43]
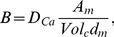
(6)where 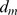 is a typical length scale. We set 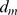 to 1 µm, which together with the diffusion constant for 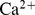, 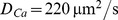 [41], [44], gives 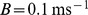.

In human 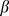-cells, GABA activates GABA*_A_* receptors, which are ligand-gated 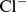 channels. We model the current carried by GABA*_A_* receptor as a passive current with the expression
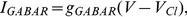
(7)where 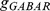 is the GABA*_A_* receptor conductance, and 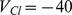 mV is the chloride reversal potential [4]. We estimate 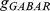 from the findings that 1 mM GABA evokes a current of 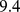 pA/pF (but with substantial cell-to-cell variation) at a holding potential of −70 mV [12], which yields a conductance of 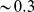 nS/pF. To simulate the changes in firing patterns evoked by lower GABA concentrations (10 or 100 µM) [12], we take into consideration the does-response curve [45] for the 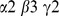 subunits, which are the most highly expressed subunits in human 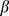-cells [12]. At 10 µM the GABA-evoked current is 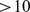-fold smaller compared to 1 mM GABA, and we set 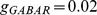 nS/pF. At 100 µM, the reduction is about 2-fold compared to 1 mM. We used 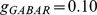 nS/pF to simulate application of 100 µM GABA.

To investigate slow electrical patterns (Fig. 6) we added a glycolytic component [11], which drives ATP levels and K(ATP) channel activity. The glycolytic subsystem can oscillate due to positive feedback on the enzyme phosphofructokinase (PFK) from its product fructose-1,6-bisphosphate (FBP). The glycolytic equations are
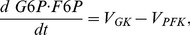
(8)
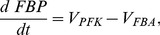
(9)
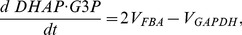
(10)where 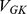 is the rate of glucokinase, which phosphorylates glucose to glucose-6-phosphate (G6P). G6P is assumed to be in equilibrium with fructose-6-phosphate (F6P), the substrate for PFK, and 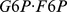 is the sum of G6P and F6P. 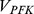 is the rate of PFK producing FBP, which is subsequently removed by fructose-bisphosphate aldolase (FBA), which produces glyceraldehyde-3-phosphate (G3P) and dihydroxyacetone-phosphate (DHAP) with rate 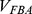. DHAP and G3P are assumed to be in equilibrium, and 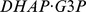 indicates their sum. Finally, G3P serves as substrate for glyceraldehyde-3-phosphate dehydrogenase (GAPDH with rate 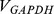), which via the lower part of glycolysis eventually stimulates mitochondrial ATP production. We introduce a phenomenological variable 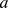 that mimics ATP levels, and is model by
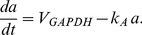
(11)The K(ATP) conductance depends inversely on 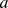, and is modeled as
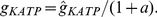
(12)Expressions and parameters are given below.

Simulations were done in XPPAUT [46] with the cvode solver, except the stochastic simulation in Fig. 1E, which was performed with the implicit backward Euler method. Computer code can be found as supplementary material, or downloaded from http://www.dei.unipd.it/ pedersen.

### Experiments

Human pancreatic islets were obtained with ethical approval and clinical consent from non-diabetic organ donors. All studies were approved by the Human Research Ethics Board at the University of Alberta. The islets were dispersed into single cells by incubation in 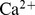 free buffer and plated onto 35 mm plastic Petri dishes. The cells were incubated in RPMI 1640 culture medium containing 7.5 mM glucose for >24 h prior to the experiments. Patch-pipettes were pulled from borosilicate glass to a tip resistance of 6–9 MΩ when filled with intracellular solution. The membrane potential was measured in the perforated-patch whole-cell configuration, using an EPC-10 amplifier and Patchmaster software (HEKA, Lambrecht, Germany). The cells were constantly perifused with heated bath solution during the experiment to maintain a temperature of 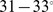
C. The extracellular solution consisted of (in mM) 140 NaCl, 3.6 KCl, 0.5 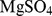, 1.5 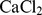, 10 HEPES, 0.5 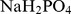, 5 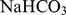 and 6 glucose (pH was adjusted to 7.4 with NaOH). The pipette solution contained (in mM) 76 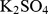, 10 KCl, 10 NaCl, 1 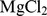, 5 HEPES (pH 7.35 with KOH) and 0.24 mg/ml amphotericin B. 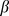-cells were identified by immunostaining (18 out of 28 cells) or by size when immunostaining was not possible (cell capacitance >6 pF, [3]). Tetrodotoxin (TTX) and 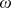-agatoxin IVA were purchased from Alomone Labs (Jerusalem, Israel), UCL-1684 was obtained from R&D Systems (Minneapolis, MN), TRAM-34 from Sigma-Aldrich (Oakville, ON, Canada). Figures with experimental responses to ion channel antagonists (Figs. 1, 3, 4 and 5) show recordings from the same cell before (ctrl) and after application of the blocker.

### Model equations and parameters

For completeness, we report all expressions and parameters of the mathematical model here. For details, please refer to the Modeling section above and the previous article [7].

The main variables, membrane potential, 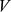, submembrane 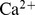, 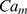, and cytosolic 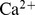, 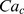, are described by
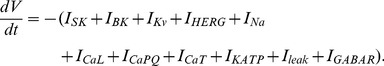
(13)
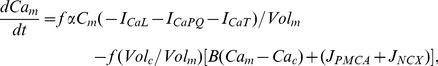
(14)
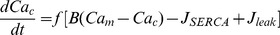
(15)

The currents are
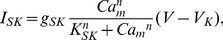
(16)
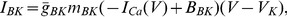
(17)
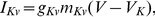
(18)
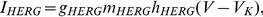
(19)
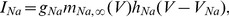
(20)
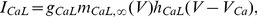
(21)
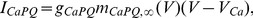
(22)
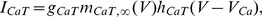
(23)
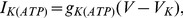
(24)
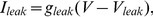
(25)
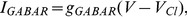
(26)where
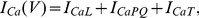
(27)and activation variables (and similarly inactivations variabels, 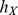, where 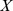 denotes the type of current) follow
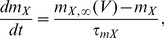
(28)where 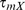 (respectively 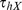) is the time-constant of activation (respectively inactivation for 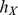), and 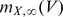 (respectively 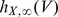) is the steady-state voltage-dependent activation (respectively inactivation) of the current. The steady-state activation (and inactivation) functions are described with Boltzmann functions,
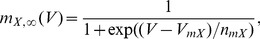
(29)except
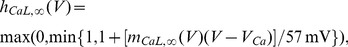
(30)for 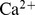 -dependent inactivation of L-type 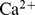 channels. The time-constant for activation of Kv-channes is assumed to be voltage-dependent [3], [7],
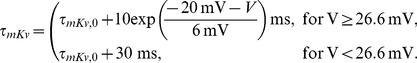
(31)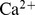 -fluxes are [40]
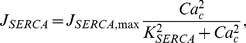
(32)
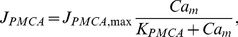
(33)
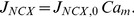
(34)Glycolysis was modeled by [11]
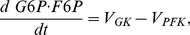
(35)
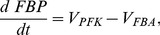
(36)
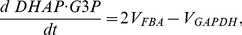
(37)which controls the electrophysiological subsystem via the “ATP-mimetic” 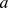 and K(ATP)-channels, as described by
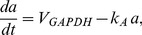
(38)
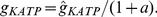
(39)Here
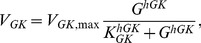
(40)
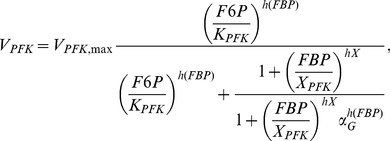
(41)
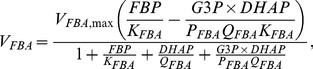
(42)
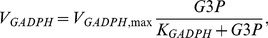
(43)where
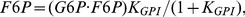
(44)
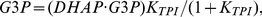
(45)
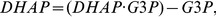
(46)and
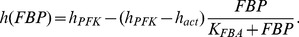
(47)Default parameters are given in Table 1.

**Table 1:** Default model parameters.

Parameter			Ref.	Parameter			Ref.
*V_K_*	−75	mV	[3]	*V_Na_*	70	mV	[7]
*V_Ca_*	65	mV	[7]	*V_Cl_*	−40	mV	[4]
*g_SK_*	0.1	nS/pF	[8]	*K_SK_*	0.57	µM	[39]
*n*	5.2		[39]				
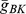	0.020	nS/pA	[3]	*τ_mBK_*	2	ms	[3]
*V_mBK_*	0	mV	[3]	*n_mBK_*	−10	mV	[3]
*B_BK_*	20	pA/pF	[3]				
*g_Kv_*	1.000	nS/pF	[3]	*τ_mKv_*_,0_	2	ms	[3]
*V_mKv_*	0	mV	[3]	*n_mKv_*	−10	mV	[3]
*g_HERG_*	0	nS/pF	+ [7], [47]				
*V_mHERB_*	−30	mV	[47]	*n_mHERG_*	−10	mV	[47]
*V_hHERG_*	−42	mV	[47]	*n_hHERG_*	17.5	mV	[47]
*τ_mHERG_*	100	ms	[48]	*τ_hHERG_*	50	ms	[47]
*g_Na_*	0.400	nS/pF	[3]	*τ_hNa_*	2	ms	[3]
*V_mNa_*	−18	mV	[3]	*n_mNa_*	−5	mV	[3]
*V_hNA_*	−42	mV	[3]	*n_hNa_*	6	mV	[3]
*g_CaL_*	0.140	nS/pF	[3]	*τ_hCaL_*	20	ms	[7]
*V_mCaL_*	−25	mV	[3]	*n_mCaL_*	−6	mV	[3]
*g_CaPQ_*	0.170	nS/pF	[3]				
*V_mCaPQ_*	−10	mV	[3]	*n_mCaPQ_*	−6	mV	[3]
*g_CaT_*	0.050	nS/pF	[3]	*τ_hCaT_*	7	ms	[3]
*V_mCaT_*	−40	mV	[3]	*n_mCaT_*	−4	mV	[3]
*V_hCaT_*	−64	mV	[3]	*n_hCaT_*	8	mV	[3]
*g_K(ATP)_*	0.010	nS/pF	[1]	*g_GABAR_*	0	nS/pF	[12], [45]
*g_leak_*	0.015	nS/pF	[1]	*V_leak_*	−30	mV	[7]
*J_SERCA_*_,max_	0.060	µM/ms	+ [40]	*K_SERCA_*	0.27	µM	[40]
*J_PMCA_*_,max_	0.021	µM/ms	[40]	*K_PMCA_*	0.50	µM	[40]
*J_leak_*	0.00094	µM/ms	[40], [49]	*J_NCX_*_,0_	0.01867	ms^−1^	[40], [49]
*f*	0.01			*Vol_c_*	1.15	×10^−12^ L	
*B*	0.1	ms^−1^		*Vol_m_*	0.1	×10^−12^ L	
*α*	5.18×10^−15^ µmol/pA/ms						
*V_GK_*_,max_	0.0000556	mM/ms	[11]	*K_GK_*	8	mM	[11]
*h_GK_*	1.7		[11]	*G*	10	mM	+
*V_PFK_*_,max_	0.000556	mM/ms	[11]	*K_PFK_*	4.0	mM	[11]
*h_PFK_*	2.5		[11]	*h_act_*	1		[11]
*X_PFK_*	0.01	mM	[11]	*h_X_*	2.5		[11]
*α_G_*	5.0		[11]				
*V_FBA,_*_max_	0.000139	mM/ms	[11]	*K_FBA_*	0.005	mM	[11]
*P_FBA_*	0.5	mM	[11]	*Q_FBA_*	0.275	mM	[11]
*V_GADPH_*_,max_	0.00139	mM/ms	[11]	*K_GADPH_*	0.005	mM	[11]
*K_GPI_*	0.3		[11]	*K_TPI_*	0.045455		[11]
*k_A_*	0.0001	ms^−1^	+	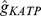	0.050	nS/pF	+

Default model parameters used in the manuscript unless mentioned otherwise. Parameter values are based on the indicated literature references (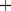 indicates adjusted parameters), see also reference [7] for a discussion of the parameters introduced there. All glycolytic parameters are taken without modification from reference [11], where a discussion of their values based on experimental data can be found. HERG channel conductance, 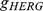, was set to zero in the present work to investigate whether SK-channels can substitute for HERG channels, e.g., in driving bursting. Based on [47], the previous version of the model [7] used 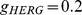 nS/pF. The conclusions presented here are not sensitive to whether or not HERG currents are included.
