# Correction: Mathematical Modeling of Heterogeneous Electrophysiological Responses in Human *β*-Cells

**DOI:** 10.1371/journal.pcbi.1003536

**Published:** 2014-02-28

**Authors:** The PLOS Computational Biology Staff

The supplementary material does not appear. Please view Software S1 and S2 below.

The supplementary material can also be downloaded here: www.dei.unipd.it/~pedersen. Please note that the link to this website appears incorrectly in the Methods section.

## Supporting Information

Software S1 XPPAUT code for Figs. 1-5.
(ODE)

Software S2 XPPAUT code for Fig. 6.
(ODE)
